# Ultrastructural study of anther parasitism of *Ficus laevigata* by *Ficophagus laevigatus* (Aphelenchoididae)

**DOI:** 10.2478/jofnem-2023-0010

**Published:** 2023-05-18

**Authors:** Robin M. Giblin-Davis, Natsumi Kanzaki, Donna S. Williams

**Affiliations:** Fort Lauderdale Research and Education Center, Department of Entomology and Nematology, University of Florida/IFAS, 3205 College Avenue, Davie, FL 33314-7799, United States of America; Kansai Research Center, Forestry and Forest Products Research Institute, 68 Nagaikyutaroh, Fushimi, Kyoto, Japan 612-0855; Department of Microbiology and Cell Science, University of Florida-IFAS, P.O. Box 10700, Gainesville, FL 32611-0700, United States of America

**Keywords:** fig, fig nematode, morphology, plant parasitism

## Abstract

Transmission electron microscopy (TEM) was used to compare the ultrastructural differences between healthy male florets (anthers) and one floret parasitized by *Ficophagus laevigatus* in late phase C syconia of *Ficus laevigata* from southern Florida. Previous light-microscopic examination of paraffin-sectioned material showed that *F. laevigatus*-infested anthers of *F. laevigata* manifested as malformed, often with aberrant pollen and hypertrophied epidermal cells closest to regions containing propagating nematodes. Female florets or fig wasp-parasitized female florets were not observed to be parasitized by nematodes. Considering that plant-feeding in the Aphelenchoididae is purportedly much less specialized than in certain groups of the Tylenchomorpha, where specialized hypertrophied feeder cells are produced in response to nematode feeding, we examined the putative induced response in this unusual aphelenchoidid system with the higher resolution afforded by TEM. TEM confirmed the expression of significant epidermal cell hypertrophy of the anther and anther filament in the presence of propagating nematodes, which was expressed as cell enlargement (2-5X), fractionation of large electron-dense stores into smaller aggregates, irregularly shaped nuclei enclosed by an elongated nuclear envelope, nucleolus enlargement, increased organelle production, and apparent metabolism with increased numbers of mitochondria, pro-plastids, and endoplasmic reticulum, as well as increased thickening of the cell walls. Pathological effects were observed in adjacent cells/tissue (e.g., anther and anther filament parenchymal cells, pollen tubes, pollen, and endothecium) with apparent diminishment as the distance from propagating nematodes increased (which was also probably affected by number of nematodes). Some TEM sections captured previously undocumented ultrastructural highlights of propagating individuals of *F. laevigatus*.

Fig wasp females *(Pegoscapus* sp.) deposit entomophilic females of the nematode *Ficophagus laevigatus* (DeCrappeo and Giblin-Davis, 2001; Davies and Bartholomaeus, 2015) into phase B sycones (small figs that are receptive to pollination with developmentally receptive female florets and immature male florets [anthers]) of *Ficus laevigata* Vahl during pollination of the fig and oviposition (Giblin-Davis et al., 1995). These nematodes (3 ± 3 per fig wasp pollinator) may be developmentally arrested during their internal ride within the fig wasp host (Giblin-Davis et al., 1995; DeCrappeo and Giblin-Davis, 2001).

Once in the fig, they leave their fig wasp host to seek out immature anthers where they mature and propagate (see morphological and morphometric differences between pre-reproductive entomophilic and mature reproductive females of *F. laevigatus* in DeCrappeo and Giblin-Davis, 2001), apparently inducing hypertrophied anther epidermal cells in the interfloral phase C fig (phase when figs grow, and fig embryos and wasp larvae develop within their respective developing female fig florets, and anthers begin to mature) (Giblin-Davis et al., 1995). This phase lasts about 30 days and results in a large population increase of hundreds of nematodes, many of which end up as the next generation of entomophilic females. These females are amassed at the appropriate location for infesting the next generation of fig wasp females as they seek out anthers to collect pollen before leaving the phase D fig (phase when anthers and pollen finish maturation, fig wasp pupae molt to adults in their galls, male fig wasps emerge to mate with female fig wasps in their galls, and female fig wasps emerge to collect pollen and leave the fig through exit holes made by the wingless male fig wasps) for a new phase B fig and the completion of one cycle of the reproductive life history of the fig, fig wasp and fig nematode (Giblin-Davis et al., 1995).

So far, *Ficus laevigata* is anomalous in having anther-exclusive nematode parasitism by *F. laevigatus* (Giblin-Davis et al., 1995; Center et al., 1999). Different florets/tissues, including anthers, are parasitized inside the developing sycones of the other five *Ficus* species that have been examined for *Ficophagus (=Schistonchus* s.l.) parasitism in the New World, but only *F. laevigata* is anther-exclusive (Center et al., 1999). All six species of *Ficophagus-infested Ficus* syconia exhibited cell hypertrophy when observed near nematodes, regardless of tissue/floret type (Center et al., 1999). However, in all the *Ficus* spp. examined, except *F. laevigata*, nematode-induced pathology was relatively minor except when associated with anthers. Figs are protogynous with female florets being receptive to pollination during phase B when male florets are not mature. This forces outcrossing by the fig wasp pollinators and means that female and male florets are at different developmental levels coinciding with *Ficophagus* infestation. Differences in pathology may accumulate in anthers more than female florets (wasp galls or fig embryos) or syconial wall tissue because of the differential effects of nematode feeding on different tissues at the different developmental levels (Center et al., 1999). Exclusive anther parasitism by nematodes could be adaptive, by increasing their potential for vertical transmission to the next generation of female pollinating fig wasps as they gather pollen at the anthers before leaving the sycone, but maladaptive to the fig by reducing viable pollen production.

Most nematodes in the family Aphelenchoididae are fungal feeders that use their stylets to pierce mycelia and suck the contents out, and some plant and insect parasites and predators in the family also feed on their food using stylets (Hunt, 1993). Feeding in the relatively few plant-parasitic members of the family Aphelenchoididae typically involves ecto- or endoparasitism and movement through stomata/direct penetration into leaf tissue (Hunt, 1993), or through pine or coconut palm stem tissue (Kanzaki and Giblin-Davis, 2018), protrusion of the stylet through the cell wall into parenchymal or mesophyll cells (Sanwal, 1959; Wallace, 1959), secretion of pharyngeal derived cellulases (Fu, 2012), withdrawal of the cytoplasm and/or organelles through the stylet into the pharynx, retraction of the stylet, and movement to a new cell for continued feeding. This type of behavior often results in the death of the injured cell and can cause tissue necrosis (Dropkin, 1969; Jones and De Waele, 1990). This same “stick, suck, and kill” method of feeding is common among the less specialized representatives of the Tylenchomorpha (Siddiqi, 2000), but there are also several lineages culminating in increased specialization with induced uni- or multinucleate feeder cells that are initiated through effector molecules secreted into selected root cells from the dorsal or subventral glands of the pharynx, e.g., Heteroderidae and Meloidogynidae (Davis et al., 2004).

Light microscopic (LM) observations of sections of paraffin-embedded figs parasitized by different *Ficophagus* and *Schistonchus* species (Aphelenchoididae) suggested that they may induce more specialized plant cell responses to propagative nematode feeding than just individual cell death and tissue necrosis (Vovlas et al., 1992; Giblin-Davis, et al., 1995; Center et al., 1999). The relative unpredictability of locating pockets of these nematode infestations while dissecting or sectioning figs (Center et al., 1999) can create a challenge for following this work up with higher resolution transmission electron microscopy (TEM) or targeted transcriptomic molecular research. However, because of the apparently unique and specific targeting of primordial anthers in *Ficus laevigata* by *Ficophagus laevigatus* (Giblin-Davis et al., 1995; Center et al., 1999) and the observation that nematode-infested anthers in freshly dissected late phase C figs appear slightly more tanned in color than healthy anthers under the dissecting microscope, we conducted this study to compare the TEM ultrastructural differences between healthy and *F. laevigatus-*infested *F. laevigata* anthers. In the process, some TEM sections arbitrarily captured a few previously undocumented ultrastructural highlights of individuals of *F. laevigatus* which were reported.

## Materials and Methods

### Transmission Electron Microscopy (TEM)

Late phase C *Ficus laevigata* figs were collected from a canal adjacent to Palm Avenue, Florida City, Dade County, Florida on March 7, 1994. Each fig was cut in half, with one half being chopped up and placed in sterilized water for >20 minutes to determine nematode presence or absence. Once determined, the other half of the fig was dissected, and whole anthers were removed for ultrastructural comparisons. Nematode-infested or uninfested (control) anthers were separately placed into 2% formaldehyde (prepared from paraformaldehyde) and 2.5% glutaraldehyde in 0.1 M cacodylate buffer at pH 7.2 and fixed overnight at 4°C. After repeated rinsing in buffer, anthers were postfixed in 2% OsO_4_ in 0.1 M cacodylate buffer at pH 7.2 for 3.5 h at 22°C. Anthers were then rinsed in water, fixed with 1% aqueous uranyl acetate, slowly dehydrated through a graded ethanol series into 100% ethanol and then into 100% acetone, and infiltrated with Spurr’s epoxy resin. Resin blocks were sectioned on a MC® MT-6000-XL ultra-microtome, and sections with silver refraction (75 nm) were picked up on copper grids with a 0.35% Formvar coating reinforced with a light carbon film. Sections were post-stained with 5% aqueous uranyl acetate and lead citrate before being viewed on a Zeiss EM-10CA® transmission electron microscope at 80 kV.

One nematode-infested anther from a nematode-infested fig and three un-infested (control) anthers from an un-infested (control) fig were examined. The anthers were sagittally (or near sagittally) sectioned, observed, and photographed for comparisons through the anther, anther filament, endothecium, and pollen tubes. Photographs were taken at 2.5K magnification in an overlapping linear series starting at the ventral side (along the anther endothecium) and running completely through to the dorsal side of the anther filament, from ensheathing perianth lobe to ensheathing perianth lobe. In all cases, the orientation of the series was standardized just below the vertical midline of the anther, where the anther filament connected to the anther, allowing for examination of the anther epidermal cells connecting to the ventral anther filament epidermal cells, because nematode-associated pathology was consistently observed in this region in LM sections (Giblin-Davis et al., 1995). A total of 90 photographs were taken using Kodak EM film 4489, including some at magnifications up to 50K for better discernment of specific targets.

The photographs were edited with Photoshop Elements 9 (Adobe) for contrast and brightness adjustments and to construct the figures. The original captures are available upon request.

## Results

**Figure 1:**
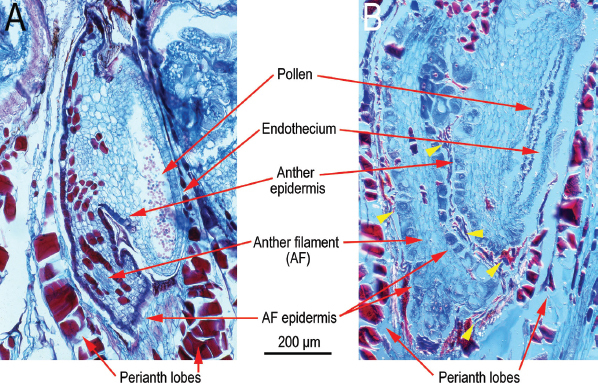
Light micrographs of near sagittal sections of *Ficus laevigata* anthers from paraffin-embedded phase C figs from Giblin-Davis et al. (1995). A: Healthy anther; B: *Ficophagus laevigatus*-infested anther. Nematodes are indicated with yellow arrowheads.

**Figure 2:**
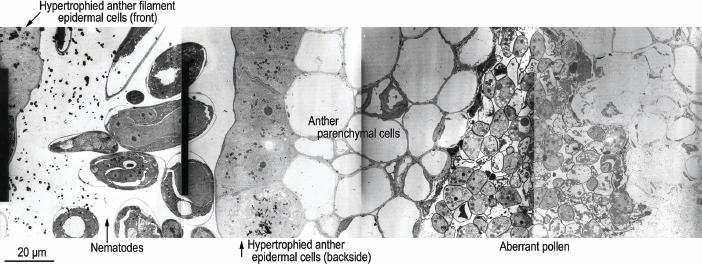
Transmission electron micrograph of near sagittal series of *Ficophagus laevigatus-*infested *F. laevigata* anther below the vertical midline showing the ventral anther filament epidermis through the dorsal anther epidermis into a pollen sac with aberrant pollen.

**Figure S1:**
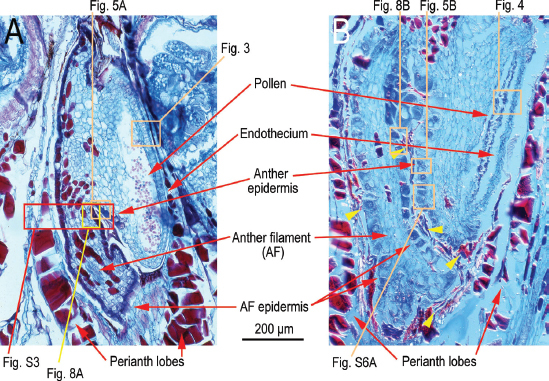
Light micrographs of near sagittal sections of *Ficus laevigata* anthers from paraffin-embedded phase C figs from Giblin-Davis et al. (1995). A: Healthy anther; B: *Ficophagus laevigatus-*infested anther. Nematodes are indicated with yellow arrowheads and colored rectangles highlight the relative location of the numbered Figures.

**Figure S2:**
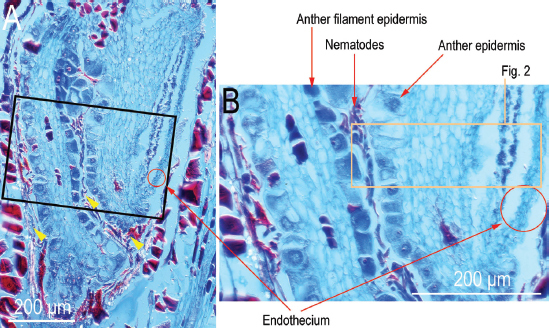
Light micrographs of near sagittal section of *Ficophagus laevigatus-*infested *Ficus laevigata* anther from paraffin-embedded phase C fig from Giblin-Davis et al. (1995). A: Hypertrophied anther and anther filament epidermis, endothecium, pollen sac, and pollen; B: Close-up of nematodes and hypertrophied anther and anther filament epidermal cells in the black box in subfigure A. Nematodes are indicated with yellow arrowheads and the colored rectangle highlights the relative location of the numbered Figure.

**Figure S3:**
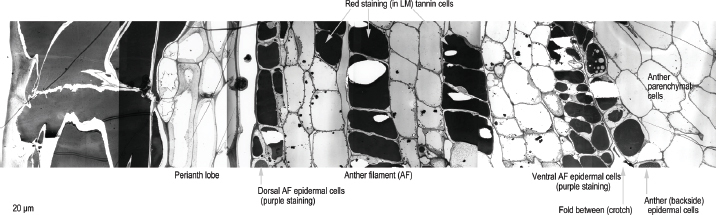
Transmission electron micrograph of near sagittal series of healthy *F. laevigata* anther from perianth lobe through the anther filament to the anther epidermis.

**Figure S4:**
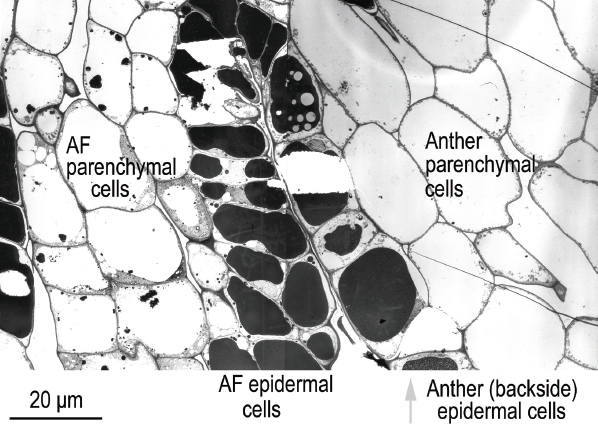
Transmission electron micrograph of near sagittal series of healthy *F. laevigata* anther below the vertical midline showing the anther epidermal cells connecting to the ventral anther filament epidermal cells.

For general orientation, light photographs of near sagittal sections of healthy (Fig. 1; Suppl. Fig. S1) and *F. laevigatus*-infested *F. laevigata* anthers (Fig. 1; Suppl. Fig. S2) from paraffin embedded sections from Giblin-Davis et al. (1995) are presented in color. Also, to help with orientation, supplementary TEM photographs of healthy (Suppl. Figs. S3,S4) and *F. laevigatus*-infested *F. laevigata* anthers (Fig. 2) are presented.

### Anther Endothecium

**Figure 3:**
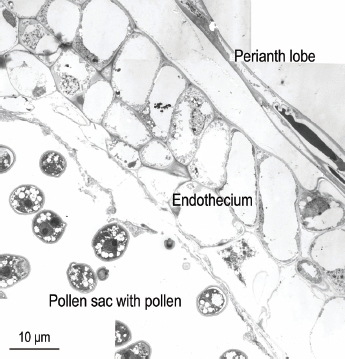
Transmission electron micrograph of near sagittal section of the ventral side of a healthy *Ficus laevigata* anther from a phase C fig, showing endothecium and pollen sac with pollen.

**Figure 4:**
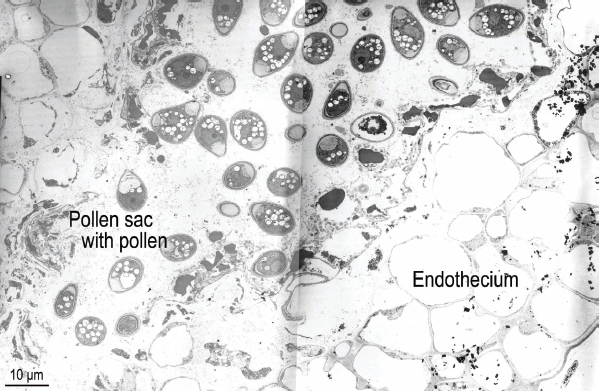
Transmission electron micrograph of near sagittal section of the ventral side of a *Ficophagus laevigatus*-infested *Ficus laevigata* anther from a phase C fig showing endothecium and pollen sac with pollen.

The endothecium in the healthy anthers appeared as described by Center et al. (1999) with LM (Fig. 1A; Suppl. Fig. S1) as a two-to-three-cell layer of highly vacuolated cells with cellular components, mostly relegated to the periphery near the cell walls, and surrounding an electron lucent vacuole (Fig. 3). The cells in the endothecium of the nematode parasitized anther in this study were similar in size and cytoplasmic density to healthy anthers (Fig. 4). Thus, the effect of nematodes occurring in the space between the anther filament and the back of the anther on the endothecium was relatively minor. Center et al. (1999) observed that the effects on the endothecium were relatively minor when compared to the anther epidermal and anther filament epidermal cells that were in direct or close contact with the nematodes/exudate; however, the endothecium lost cell integrity as the densities of nematodes increased (Fig. 2; Suppl. Figs. S1, S2).

### Anther Epidermis

**Figure 5:**
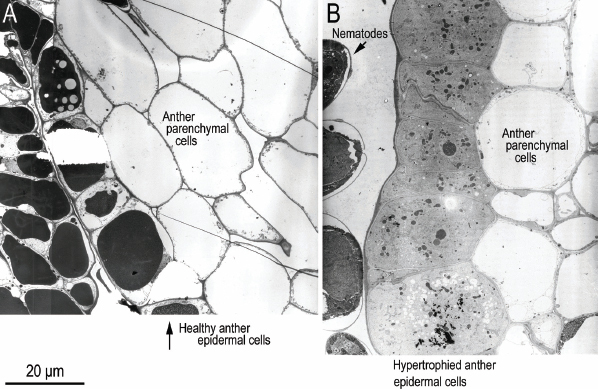
Transmission electron micrographs of near sagittal sections of *Ficus laevigata* anthers. A: Healthy anther; B: *Ficophagus laevigatus-*infested anther showing the effects of nematodes on anther epidermal and parenchymal cells in the region where the anther epidermal cells connect to the ventral anther filament epidermal cells.

**Figure 6:**
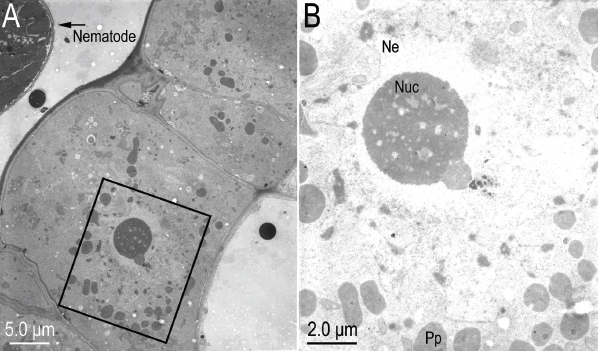
Transmission electron micrographs of near sagittal sections of *Ficophagus laevigatus-*infested *Ficus laevigata* anther amplifying the area shown in Fig. 5B. A: Close-up of hypertrophied anther epidermal cells; B: Magnification of the area in the black box to show the irregular indentations of the nuclear envelop (NE), nucleolus (Nuc) and putative pro-plastids (Pp).

**Figure 7:**
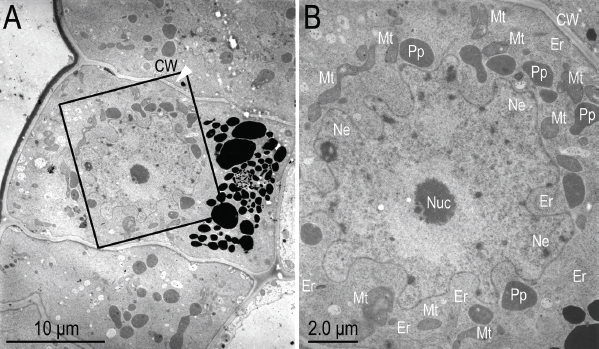
Transmission electron micrographs of near sagittal sections of *Ficophagus laevigatus-*infested *Ficus laevigata* anther amplifying the area shown in Fig. 5B. A: Close-up of hypertrophied anther epidermal cells; B: Magnification of the area in the black box to show the thickened cell walls (CW), increased abundance of endoplasmic reticulum (ER) and mitochondria (Mt), enlarged nucleus with irregular indentations of the nuclear envelop (NE), nucleolus (Nuc), and presence of putative pro-plastids (Pp).

**Figure S5:**
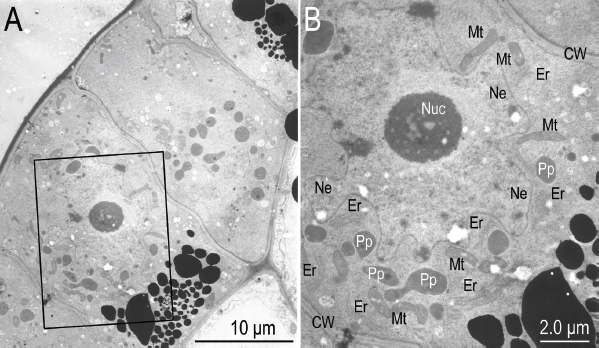
Transmission electron micrographs of near sagittal sections of *Ficophagus laevigatus-*infested *Ficus laevigata* anther amplifying the area shown in Fig. 5B. A: Close-up of hypertrophied anther epidermal cells; B: Magnification of the area in the black box to show the thickened cell walls (CW), increased abundance of endoplasmic reticulum (ER) and mitochondria (Mt), enlarged nucleus with irregular indentations of the nuclear envelop (NE), nucleolus (Nuc), and presence of putative pro-plastids (Pp).

**Figure S6:**
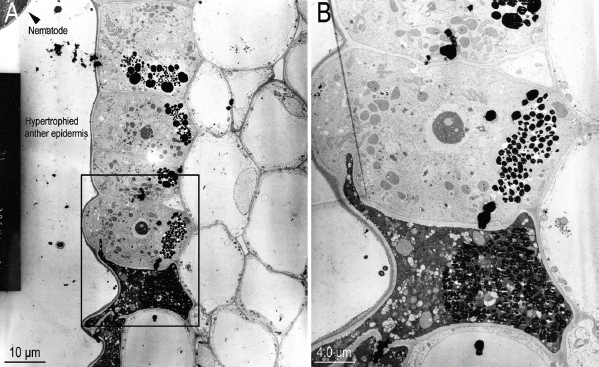
Transmission electron micrographs of near sagittal section of *Ficophagus laevigatus-*infested *Ficus laevigata* anther showing the effects of nematodes on anther epidermal and parenchymal cells in the region below where the anther epidermal cells connect to the ventral anther filament epidermal cells. A: Whole section; B: Close-up of hypertrophied anther epidermal cells in the black box in subfigure A.

Sagittal sections of the healthy anther epidermis comprised a single layer of cells with large central storage vacuoles filled with very electron-dense and darkly staining material and cellular components that were mostly arranged on the periphery near the cell walls (Fig. 5A; Suppl. Figs. S3,S4). This epidermal layer bounded a lightly staining multilayer of parenchymal cells that surrounded the pollentubes/tapetum. In the nematode-infested anther, this single layer of epidermal cells was hypertrophied and 2 to 5 times the size of the cells in the healthy epidermis with thickened cell walls (Figs. 2, 5B). Each hypertrophied cell had an enlarged and often irregularly shaped nucleus with an enlarged nucleolus, and were metabolically active as indicated by increased numbers of mitochondria and what appeared to be pro-plastids as well as endoplasmic reticulum. The darkly stained central storage vacuole had broken up into many smaller darkly stained globs, or less electron-dense granular cytoplasm of much higher volume in the enlarged cells (Figs. 6, 7; Suppl. Fig. S5). The bounded parenchymal cells were also slightly enlarged and had slightly thickened cell walls (Fig. 5B; Suppl. Fig. S6).

### Anther Filament

**Figure 8:**
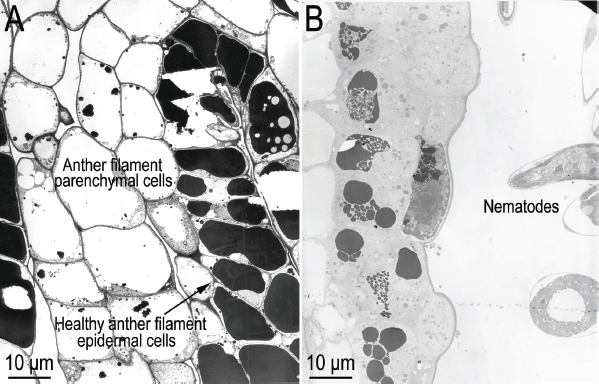
Transmission electron micrographs of near sagittal sections of *Ficus laevigata* anthers. A: Healthy anther; B: *Ficophagus laevigatus-infested* anther showing the effects of nematodes on anther filament epidermal and parenchymal cells in the region where the anther epidermal cells connect to the ventral anther filament epidermal cells.

**Figure 9:**
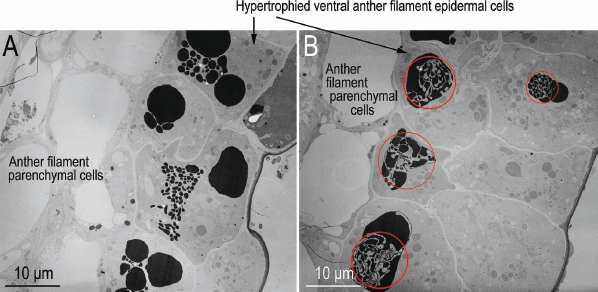
Transmission electron micrographs of near sagittal sections of *Ficophagus laevigatus-*infested *Ficus laevigata* anther showing the effects of nematodes on ventral anther filament epidermal and parenchymal cells in two different areas. Red circles denote regions where dissipation of electron dense vacuolar material was occurring.

**Figure 10:**
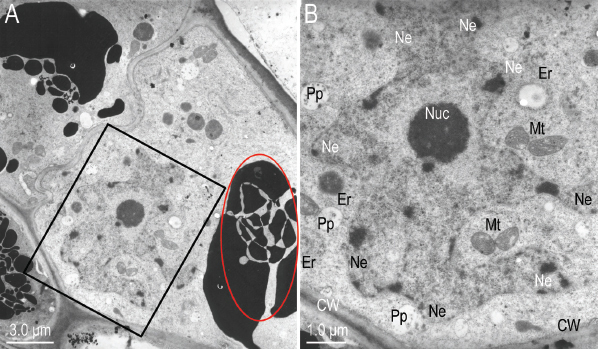
Transmission electron micrographs of near sagittal sections of *Ficophagus laevigatus-*infested *Ficus laevigata* anther. A: The effects of nematodes on ventral anther filament epidermal cells; B: Close-up of hypertrophied ventral anther filament epidermal cell in the black box in subfigure A to show the thickened cell walls (CW), increased abundance of endoplasmic reticulum (ER) and mitochondria (Mt), enlarged nucleus with irregular indentations of the nuclear envelop (NE), nucleolus (Nuc), and presence of putative pro-plastids (Pp). Red circle in subfigure A denotes region where dissipation of electron dense vacuolar material was occurring.

Sagittal sections of healthy ventral anther filament epidermis near the connection to the backside of the anther showed a single to double (rarely triple) layer of cells that were smaller or near to the same size as the adjacent anther epidermal cells with similarly large central storage vacuoles, filled with very electron-dense and darkly stained material, and with cellular components arranged mostly at the periphery near the cell walls (Fig. 8A; Suppl. Figs. S3,S4). This layer of cells bounded a light-staining multilayer of parenchymal cells that was marbled with short runs of 3-7 larger, more squared-looking cells with similarly large central storage vacuoles, filled with very electron-dense and darkly stained material, and with cellular components mostly present at the periphery near the cell walls (Fig. 1; Suppl. Fig. S3) (these latter cells were equivalent to the safranin-rich red-staining cells in the filaments of healthy and nematode-infested anthers observed with LM by Giblin-Davis et al.,1995). Ventral anther filament epidermal cells near the propagating nematodes were similar to the adjacent anther epidermal cells with hypertrophy of the single or multiple layers with increases in cell size of 2-5x, a decentralization and dissipation of the dense material in the storage vacuoles creating a more granular-looking cytoplasm, enlargement of the irregularly bounded nucleus and spherical nucleolus, thickening of cell walls, and an apparent increase in cell metabolism (Figs. 8B-10). Mobilization of electron-dense vacuolar material appeared to be occurring in some epidermal cells (Figs. 9B, 10A).

**Figure 11:**
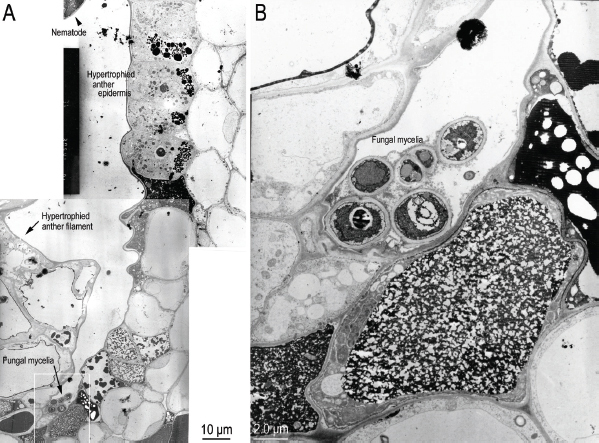
Transmission electron micrographs of near sagittal sections of *Ficophagus laevigatus-*infested *Ficus laevigata* anther, showing the effects of nematodes on anther and anther filament epidermal cells in a region where the anther epidermal cells connect to the ventral anther filament epidermal cells and where a few strands of fungal mycelia were observed. A: Whole section; B: Close-up of fungal mycelia from white box in subfigure A.

We also observed a few strands of fungal mycelium in the area between the anther and anther filament but found nothing that could reasonably have nourished the population of propagating nematodes or that could explain induction of the pathological effects observed in association with the nematodes (Fig. 11).

### Anther Pollen Tubes

Healthy pollen was observed in pollen tubes in each of the three un-infested anthers that were sectioned and photographed (Fig. 3). In addition, relatively healthy pollen was observed in one of the two pollen tubes observed in the nematode-infested anther (Fig. 4). The pollen tube expressing the most pathology was closest to the hypertrophied anther epidermal cells nearest nematodes in the sectioned anther (Fig. 2), suggesting a proximity-based systemic reaction to the nematode infestation. This supports the observations of Giblin-Davis et al. (1995) from LM observations of paraffin-sectioned material where “pollen production often appeared normal in one or more pollen sacs of lightly infested male florets. However, in heavily infested male florets, the four pollen sacs were completely deformed. The tapetum was sometimes necrotic and (or) disorganized.”

### Nematodes

**Figure 12:**
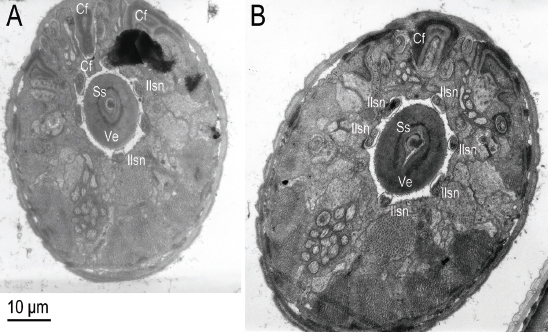
Transmission electron micrographs A, B: Two near serial and transverse-sections of the head of *Ficophagus laevigatus* within a *Ficus laevigata* anther showing the stylet shaft (Ss) tip within the vestibule (or vestibule extension) (Ve) which was ringed by an electron lucent material (or space) and the six equally spaced inner labial sensilla nerve (ilsn) pairs through the base of the cephalic framework (Cf).

**Figure 13:**
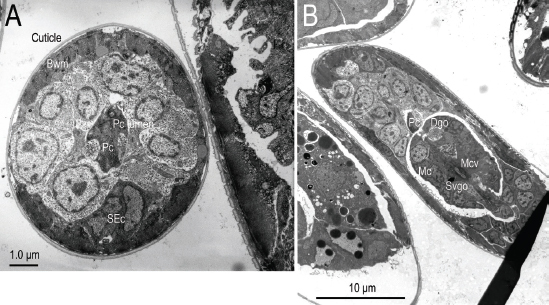
Transmission electron micrographs of the anterior regions of *Ficophagus laevigatus* within a *Ficus laevigata* anther. A: A near transverse-section of body wall muscle (Bwm), procorpus (Pc), procorpus lumen, and secretory excretory cells (SEc); B: One near sagittal-mid-section of the anterior region showing the dorsal gland orifice (Dgo), metacorpus (Mc), metacorpal valve (Mcv), procorpus (Pc), and subventral gland orifice (Svgo).

**Figure 14:**
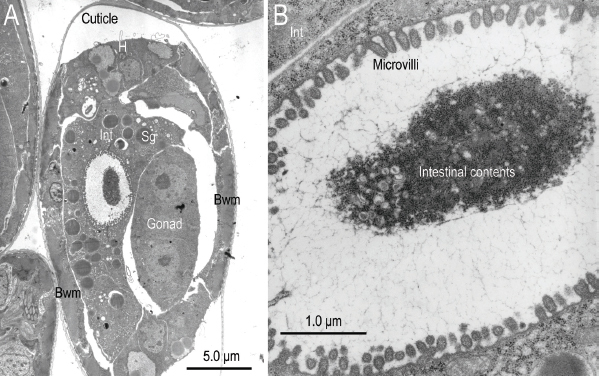
Transmission electron micrographs of the mid-region of Ficophagus laevigatus within a Ficus laevigata anther. A: A near transverse-section of body wall muscle (Bwm), hypodermis (H), intestine (Int), and secretory globules (Sg); B: Close-up of the intestinal lumen from subfigure A showing microvilli and intestinal contents.

We arbitrarily sectioned through more than ten *F. laevigatus* in the nematode-infested anther between the anther epidermal cells and the ventral anther filament epidermal cells, revealing several ultrastructural snapshots of their morphology. Going from anterior to posterior of *F. laevigatus*, we observed: 1) two near transverse sections of the head showing the stylet shaft tip with a lateral aperture (with secretion?) within the vestibule (or vestibule extension?) which was ringed by an electron lucent material (or space) and the six equally spaced inner labial sensilla nerve (ilsn) pairs through the base of the cephalic framework (Fig. 12); 2) one near transverse section of the anterior region showing the procorpus, procorpus lumen, and secretory excretory cells (Fig. 13A); 3) one near mid-sagittal section of the anterior region showing the procorpus and metacorpus (Fig. 13B); and 4) two near transverse sections of the female (?) midbody showing the intestinal syncytium with a close-up of microvilli and the digested food within as well as the gonad (Fig. 14).

### Perianth Lobes

The ultrastructure of the perianth lobes was very similar in both healthy and infested anthers (Fig. 1).

## Discussion

In comparing nematode-induced plant pathology using LM versus TEM, the adage “A picture is worth a thousand words” comes quickly to mind. We were able to corroborate the findings of Giblin-Davis et al. (1995) and Center et al. (1999) concerning the association of highly enlarged hypertrophied anther and anther filament epidermal cells in phase C figs of *Ficus laevigata* that were directly associated with propagating *Ficophagus laevigatus*. The greater resolution afforded by TEM showed how these cells responded to nematode infestation by increasing metabolic activity and transforming into something analogous to giant “feeder” cells. Unfortunately, we have not observed the nematodes in the act of penetrating or feeding on any of these cells, either with LM or TEM. The gut contents of TEM-sectioned *F. laevigatus* was consistent with what might be expected after digesting the contents of the cytoplasm of hypertrophied anther epidermal cells, but this was not conclusive. Clearly, the sectioned nematodes looked healthy, and previous reports have documented large population increases inside each infested anther (i.e., a conservative estimate of 20–275 nematodes per anther) (Giblin-Davis et al., 1995). The thicker cell walls that were observed in hypertrophied anther and anther filament epidermal cells in response to the nematode infestation could be indicative of a hardening that allows for sustained feeding by nematodes with their long and very thin stylet conus. We did not observe the exudate that was reported in heavily infested anthers in LM histopathology of *F. laevigatus* infested *F. laevigata* anthers (Giblin-Davis et al., 1995; Center et al., 1999) but this could have been because of the relatively modest nematode infestation in the TEM-sectioned anther in this study. In addition, TEM made it easier to visualize the seemingly dose-dependent proximity effect of nematode presence and the resultant pathological effects on pollen development (Fig. 2).

Although everything points to plant parasitism via population feeding of *F. laevigatus* in infested *F. laevigata* anthers by intermittent sucking or cytoplasmic bleeding from hypertrophied cells, we have not found any direct proof of this. However, this TEM study does show that the system is tractable for future experiments using transcriptomics to help elucidate what might be going on. We know that in each half of a late phase C fig of *F. laevigata* that is determined to be nematode positive by assaying the other half, we should have about 66 florets, of which about 13 should be male (anthers) and about 6 should be nematode-infested (Giblin-Davis et al., 1995). Given that we can use the increased tan coloration of infested anthers for more accurate selection, we should be able to select these into an RNA preservative and do transcriptomic comparisons with un-infested anthers from nematode infested sycones versus same-aged anthers from nematode-free sycones (control) to see what nematode and plant genes are up or down regulated for comparisons. Nematodes could also be harvested from the anther tissue for transcriptomics in order to better understand their contribution to the transciptome. In addition, future attempts should be made at in vitro culture of *F. laevigatus* using fig floret tissue culture to further elucidate the nature of plant parasitism in this system.

## Acknowledgements

The authors sincerely thank Barbara J. Center for her assistance during this study and previous support of RGD and NK throughout her outstanding career in support of nematology and life sciences at the University of Florida/IFAS, Fort Lauderdale Research and Education Center.
